# Miodesin^TM^ Positively Modulates the Immune Response in Endometrial and Vaginal Cells

**DOI:** 10.3390/molecules27030782

**Published:** 2022-01-25

**Authors:** Carlos Rocha Oliveira, Hudson Polonini, Maria Cristina Marcucci, Rodolfo P. Vieira

**Affiliations:** 1 School of Medicine, Anhembi Morumbi University, Avenida Deputado Benedito Matarazzo 6070, Sao Jose dos Campos 12231-280, SP, Brazil; 2 Postgraduate Program in Biomedical Engineering, Department of Science and Technology, Federal University of Sao Paulo (UNIFESP), Rua Talim, 330, Vila Nair, Sao Jose dos Campos 12231-280, SP, Brazil; 3 Fagron BV, Fascinatio Boulevard 350, 3065 WB Rotterdam, The Netherlands; hudson.polonini@fagron.com or; 4 Department of Biosciences and Oral Diagnosis, Institute of Science and Technology, Universidade Estadual Paulista-UNESP, Sao Jose dos Campos 12231-280, SP, Brazil; cris.marcucci@yahoo.com.br; 5 Post-graduation Program in Sciences of Human Movement and Rehabilitation, Federal University of Sao Paulo (UNIFESP), Avenida Ana Costa 95, Santos 11060-001, SP, Brazil; rodrelena@yahoo.com.br; 6 Post-Graduation Program in Human Movement and Rehabilitation, Unievangelica, Avenida Universitária KM 3,5, Anapolis 75083-515, GO, Brazil; 7 Brazilian Institute of Teaching and Research in Pulmonary and Exercise Immunology (IBEPIPE), Rua Pedro Ernesto 240, Sao Jose dos Campos 12231-280, SP, Brazil

**Keywords:** Miodesin^TM^, anti-inflammatory, endometriosis, leiomyoma, chemokines

## Abstract

Endometriosis presents high prevalence and its physiopathology involves hyperactivation of endometrial and vaginal cells, especially by bacteria. The disease has no cure and therapies aiming to inhibit its development are highly desirable. Therefore, this study investigated whether Miodesin^TM^ (10 µg/mL = IC_80_; 200 µg/mL = IC_50_), a natural compound constituted by *Uncaria tomentosa*, *Endopleura uchi*, and astaxanthin, could exert anti-inflammatory and anti-proliferative effects against Lipopolysaccharides (LPS) stimulation in endometrial and *Candida albicans* vaginal cell lines. VK2 E6/E7 (vaginal) and KLE (epithelial) cell lines were stimulated with *Candida albicans* (1 × 10^7^ to 5 × 10^7^/mL) and LPS (1 μg/mL), respectively. Miodesin^TM^ inhibited mRNA expression for Nuclear factor kappa B (NF-κB), ciclo-oxigenase 1 (COX-1), and phospholipase A2 (PLA2), beyond the C–C motif chemokine ligand 2 (CCL2), CCL3, and CCL5 in VK2 E6/E7 cells (*p* < 0.05). In addition, the inhibitory effects of both doses of Miodesin^TM^ (10 µg/mL and 200 µg/mL) resulted in reduced secretion of interleukin-1β (IL-1β), IL-6, IL-8, tumor necrosis factor α (TNF-α) (24 h, 48 h, and 72 h) and CCL2, CCL3, and CLL5 (*p* < 0.05) by VK2 E6/E7 cells. In the same way, COX-1 Miodesin^TM^ inhibited LPS-induced hyperactivation of KLE cells, as demonstrated by reduced secretion of IL-1β, IL-6, IL-8, TNF-α (24 h, 48 h, and 72 h) and CCL2, CCL3, and CLL5 (*p* < 0.05). Furthermore, Miodesin^TM^ also inhibited mRNA expression and secretion of matrix metalloproteinase-2 (MMP-2), MMP-9, and vascular endothelial growth factor (VEGF), which are key regulators of invasion of endometrial cells. Thus, the study concludes that Miodesin^TM^ presents beneficial effects in the context of endometriosis, positively affecting the inflammatory and proliferative response.

## 1. Introduction

Endometriosis is estimated to affect around 10% of the female population in reproductive age, reaching 20–90% in those with pelvic pain and/or infertility. It currently has no cure, and it is not possible to predict whether it will have relapses. Many patients report changes in quality of life, mainly due to pain and emotional problems related to infertility, among other factors [1].

This condition can be understood as a chronic and estrogen-dependent disease, characterized by the growth of endometrial tissue outside of the uterine cavity. Although its etiology remains elusive, a growing body of evidence indicates a key role of the immunological and inflammatory component involved in its development and progression [2]. The pro-inflammatory cascade in endometriosis triggers changes in cell proliferation, adhesion, and migration of endometrial tissue, which are fundamental for the success of ectopic implantation [3]. These events are also essential for induction of endometrial lesions in the peritoneal cavity, which have a key role of matrix metalloproteinase (MMP), such as MMP-2 [4], MMP-9 [5], and VEGF [6]. Furthermore, such a process occurs due to an orchestration by nuclear factor kappa beta (NF-kβ) [7]. NF-kβ has been proven to be essential in the physiopathology of endometriosis and is directly related to invasion of endometrial cells [7]. Cyclooxygenase enzyme (COX), in its two isoforms COX-1 and COX-2, has been described to centrally regulates the inflammatory process in endometriosis [8]. In addition, phospholipase A2 (PLA2) which possesses a potent pro-inflammatory property has been found to be increased in the peritoneal fluid of patients with endometriosis [9]. More recently, a group of researchers demonstrated increased expression of PLA2 in endometriotic ovarian cysts and positive correlations with the progesterone receptor (PR-B) and estrogen receptor (Erβ) [10].

Additionally, endometriosis and leiomyoma can coexist and trigger a significant number of symptomatic patients, which may explain the high pain scores reported by some patients with leiomyoma [11]. In this group of patients, the development of medical therapies that could ameliorate pelvic pain, abnormal bleeding, and reduce uterine volume would be of utmost relevance [12]. Leiomyomas are benign gynecological tumors, composed of monoclonal cells arising from the myometrium that have specific genetic mutations and are highly sensitive to the effects of steroid hormones, characterized by increased expression of estrogen and progesterone receptors when compared to the normal myometrium [13]. Treatment for both conditions includes surgery and medication, including pain medications, such as non-steroidal anti-inflammatory agents or hormonal therapy, such as gonadotropin-releasing hormone (GnRH) agonists and androgens [14].

Regarding the drug treatment of leiomyoma, gonadotropin releasing hormone analogs (GnRHa) are agents approved by the Food and Drug Administration (FDA) for temporary preoperative use to reduce leiomyoma-related blood loss and correct the resulting iron deficiency anemia. Other agents, such as selective estrogen receptor modulators, antiprogestins, aromatase inhibitors, carbegoline, danazol, and gestrinone, have been evaluated for the treatment of uterine leiomyomas with varying degrees of success [15].

Through searches for new options to treat endometriosis and leiomyoma, a natural alternative called Miodesin^TM^ was developed. It is a natural, phytotherapeutic anti-inflammatory food supplement developed to prevent and treat subclinical inflammation and its harmful effects to the body, reducing the risk of chronic degenerative diseases of inflammatory origin. Miodesin^TM^ is a proprietary blend of three natural ingredients: *Uncaria tomentosa*, *Endopleura uchi* and *Haematococcus pluvialis*. More than a simple mix of extracts, the starting materials are processed together through a special technological process responsible for its distinctive chemical composition: it has a minimum of 2.7% of alkaloids (in terms of bergenin), 0.54% of tanins (in terms of pyrogallol) and 0.24% of astaxanthin. *U. tomentosa* (Willd.) DC. (Rubiaceae) (popular name: cat’s claw) is a species native to the forests of Central and South America, used for thousands of years by the peoples of the Peruvian and Brazilian Amazon—the first register of its use dates back to the Incas. It possesses indole and oxindole alkaloids, glucosinolates, flavonoids, sterols, carbolines, and polyunsaturated fatty acids, which confer immunostimulant, antioxidant and anti-inflammatory activities [16]. *E. uchi* (Huber) Cuatrec. (Humiriaceae) (popular name: uxi) is a tree found throughout the entire Brazilian part of the Amazon basin. Its major constituents are phenolic compounds, specially bergenin [17]. Traditional medicinal applications of the stem bark of *E. uchi* are diverse and include anti-inflammatory, antioxidant, antibacterial, and antifungal activities [17,18,19,20]. *H. pluvialis* (Chlorophyceae, Volvocales) is a unicellular freshwater microalga found in temperate regions around the world and considered to be the main source of astaxanthin (3,3′-dihydroxy-β-carotene-4,4′-dione) [21]. Astaxanthin is often referred as a “super-antioxidant” molecule, because of its capacity to reduce free radicals and oxidative stress: 65 times greater than vitamin C, 54 times more than β-carotene, and 100 times more than α-tocopherol [22]. Additionally, the molecule also presents immunomodulating activity [23,24,25].

Miodesin^TM^ has previously been shown to exert anti-inflammatory effects in reducing pelvic pain in patients with endometriosis and leiomyoma, supporting the role of naturally occurring anti-inflammatory medications in the treatment of this disease [12,26,27]. However, the underlying mechanisms involved in such beneficial effects remains unexplored.

Therefore, the present study investigated the effects of Miodesin™ on activation, proliferation, and cytotoxicity of key cells (vaginal and endometrial) involved in endometriosis and leiomyoma.

## 2. Results

### 2.1. Effects of Miodesin^TM^ on Cell Line Viability

**Figure 1:**
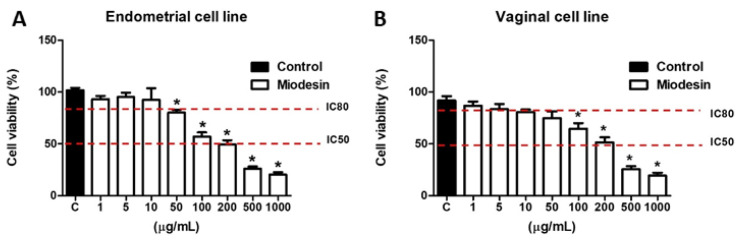
Effects of Miodesin^MT^ on endometrial (KLE) (**A**) and vaginal cell (VK2 E6/E7) (**B**) lines. Cells were treated with different concentrations of Miodesin^MT^ for 24 h. The red dotted line represents the IC_80_ and IC_50_ point. The IC_80_ was defined as the study test concentration (10 µg/mL) while IC_50_ was defined as the study test concentration (200 µg/mL). Date shown are representative of three replicates in at least three independent experiments. * *p* < 0.05 indicates significant differences (ANOVA). Values are expressed as means ± SEM.

Different concentrations of Miodesin^TM^ were tested (0–1000 µg/mL) and the IC_50_ value (200 µg/mL) and IC_80_ value (10 µg/mL) were defined as the study dose in endometrial (Figure 1A) and vaginal (Figure 1B) cell lines.

### 2.2. Pro-Inflammatory Response Induced by Candida Albicans in Vaginal Cell Line VK2 E6/E7 Is Inhibited by Miodesin^TM^

Table 1 shows the secretion of interleukins (IL-6, IL-8, IL-1β, and TNF-α) by vaginal cell line (VK2 E6/E7). Miodesin™ (10 µg/mL and 200 µg/mL) significantly reduced *Candida albicans*-induced release of pro-inflammatory interleukins (IL-6, *p* < 0.05; IL-8, *p* < 0.05; IL-1β, *p* < 0.05, and TNF-α, *p* < 0.05). Similarly, Miodesin™ (10µg/mL and 200µg/mL) also reduced *Candida albicans*-induced release of chemokines (CCL2, *p* < 0.05; CCL3, *p* < 0.05), but not for (CCL5/RANTES, *p* > 0.05), which are shown in Table 2. In summary, such results clearly show the anti-inflammatory effects of Miodesin™ on vaginal cells, not only by inhibiting the release of pro-inflammatory cytokines, but also inhibiting the release of chemokines.

### 2.3. Expression of Cytokine and Inflammatory Mediators at mRNA Levels in Vaginal Cell Line (VK2 E6/E7)

**Figure 2:**
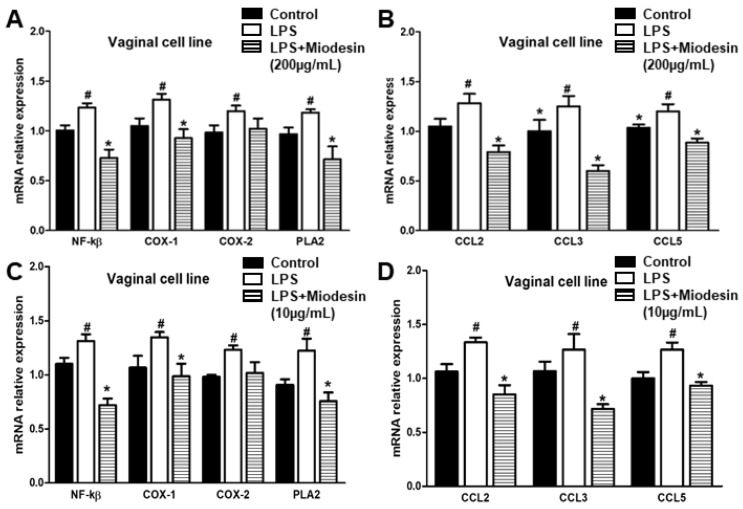
Effects of Miodesin^TM^ (200 µg/mL and 10 µg/mL) on the expression of mRNA of NF-κβ, inflammatory enzymes and chemokines from endometrial (VK2 E6/E7) cells in presence or absence of LPS. (**A**) mRNA levels were significantly reduced (* *p* < 0.05) for NF-kβ, COX-1 and PLA2, except for COX-2 mRNA levels. (**B**) The mRNA levels of the chemokines CCL2/MCP-1, CCL3/MIP-1α, and CCL5/RANTES were significantly reduced. (**C**) mRNA levels were significantly reduced (* *p* < 0.05) for NF-kβ, COX-1, COX-2, and PLA2. (**D**) mRNA levels were significantly reduced (* *p* < 0.05) for CCL2, CCL3, and CCL5. mRNA levels were determined using real-time RT-PCR. Values are expressed as means ± SEM. (#) *p* < 0.05 LPS vs. control cells. (*) *p* < 0.05 Miodesin^TM^ vs. LPS.

We also investigated the effects of Miodesin^TM^ (10 µg/mL and 200 µg/mL) on the mRNA expression of transcription factor, inflammatory mediators, and chemokines in VK2 E6/E7 cells. Similar results were found for both doses of Miodesin^TM^ (Figure 2A,B, 200 µg/mL; Figure 2C,D, 10 µg/mL), resulting in reduced expression of total NF-κβ transcription factor, inflammatory mediators, and chemokines at mRNA levels. These results indicate that Miodesin^TM^ significantly suppressed the expression of NF-κβ transcription factor and also that of enzymes that participate in the arachidonic acid cascade (inflammatory mediators), such as COX-1 and PLA2, as well as reducing chemokine expression, CCL2/MCP-1, CCL3/MIP-1α, and CCL5/RANTES.

### 2.4. Miodesin^TM^ Down-Regulates LPS-Induced Inflammatory Cytokine and Chemokine Release by Endometrial Cell Line (KLE)

**Figure 3:**
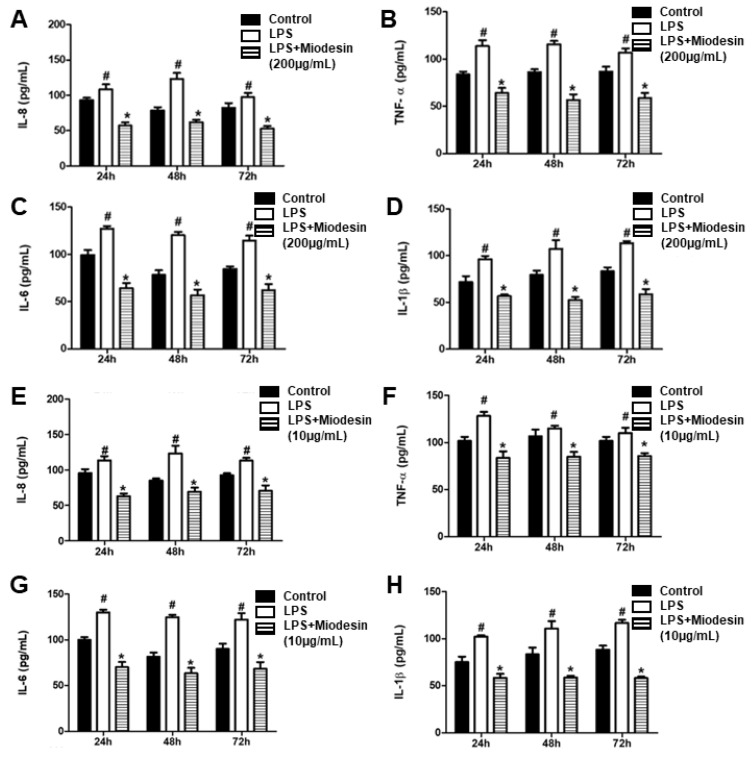
Miodesin^MT^ regulates LPS-induced release of inflammatory cytokines. Endometrial cells (KLE cells) were pretreated with Miodesin^TM^ (200 µg/mL and 10 µg/mL) for 2 h and treated with LPS (1 µg/mL) for 24 h. In subfigures (**A**–**D**), the dose of Miodesin^TM^ was 200ug/mL, and in subfigures (**E**–**H**) the dose of Miodesin^TM^ was 10ug/mL. The effect of Miodesin^TM^ on decreasing release of interleukines evaluated was determined by ELISA. Values are expressed as means ± SEM. Values are expressed as means ± SEM. (#) *p* < 0.05 LPS vs. control (non-treated cells). (*) *p* < 0.05 Miodesin^TM^ vs. LPS.

Secretion of pro-inflammatory cytokines IL-8 (Figure 3A), TNF-α (Figure 3B), IL-6 (Figure 3C), and IL-1β (Figure 3D) was significantly reduced in endometrial cells pre-treated with Miodesin^TM^ (200 µg/mL). In addition, the effects of Miodesin™ over time (24, 48, and 72 h) was maintained, reinforcing the anti-inflammatory effects of Miodesin™ in the time-course studied. Similarly, lower dose of Miodesin™ (10 µg/mL) also resulted in decreased secretion of pro-inflammatory cytokines IL-8 (Figure 3E), TNF-α (Figure 3F), IL-6 (Figure 3G), and IL-1β (Figure 3H).

Table 3 shows the results obtained with endometrial cells (KLE) stimulated with LPS which were pre-treated with 200 µg/mL and 10 µg/mL of Miodesin^TM^. Miodesin^TM^ significantly reduced the chemokine release (CCL2/MCP-1, *p* < 0.05; CCL3/MIP-1α, *p* < 0.05; CCL5/RANTES, *p* < 0.05). Again, such results clearly show the anti-inflammatory effects of Miodesin™ on endometrial cells, not only by inhibiting the release of pro-inflammatory cytokines, but also by inhibiting the release of chemokines.

### 2.5. Miodesin^TM^ Regulates MMP-2, MMP-9, and VEGF in Endometrial Cells

**Figure 4:**
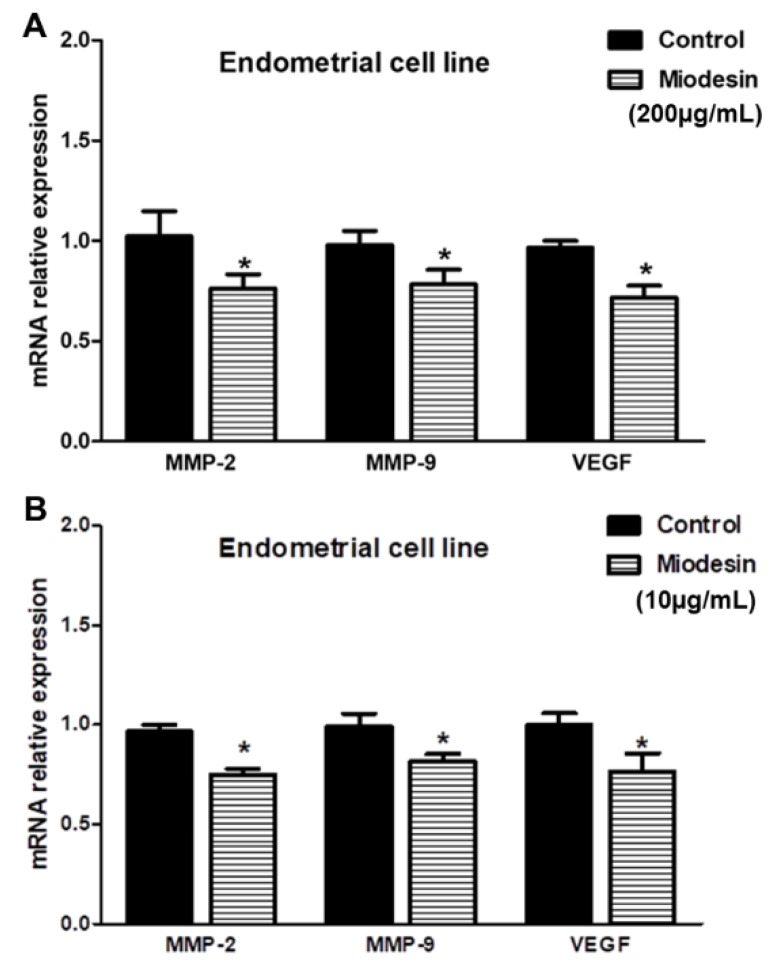
Effects of Miodesin^TM^ (**A**), 200 µg/mL; (**B**), 10 µg/mL on the expression of mRNA of MMP-2, MMP-9, and VEGF in KLE cells. mRNA levels were significantly reduced for MMP-2, MMP-9, and VEGF. KLE cells were treated with Miodesin^TM^ (200 µg/mL and 10 µg/mL) for 24 h. Real-time RT-PCR was performed to measure the mRNA levels of MMP-2, MMP-9, and VEGF in KLE cells. Glyceraldehyde-3-phosphate dehydrogenase (GADPH) was used as an internal control. Data are presented as the means ± SEM of three independent experiments. (*) *p* < 0.05.

**Figure 5:**
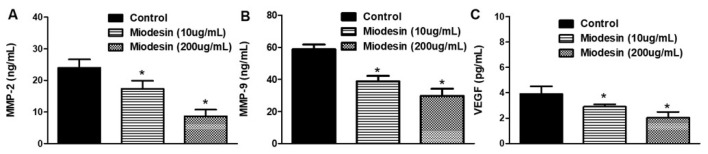
Effects of Miodesin^TM^ (200 µg/mL and 10 µg/mL) on the production of MMP2, MMP9, and VEGF. Following treatment with Miodesin^TM^ (200 µg/mL) for 24 h. ELISA analysis was performed to determine the levels of MMP2 (**A**), MMP9 (**B**), and VEGF (**C**) in the cell culture supernatant. Data are presented as the means ± SEM of three independent experiments. (*) *p* < 0.05.

To investigate whether Miodesin^TM^ regulates MMP-2, MMP-9, and VEGF expression at transcriptional level, reverse transcription polymerase chain reaction (RT-PCR) was performed. As shown in Figure 4, Miodesin^TM^ (Figure 4A, 200 µg/mL; Figure 4B, 10 µg/mL) significantly inhibited the expression of MMP-2, MMP-9, and VEGF mRNA in KLE cells. Such results at mRNA levels were confirmed at protein levels, since Miodesin^TM^ (200 µg/mL and 10 µg/mL) treatment led to significant decreases in MMP-2, MMP-9, and VEGF levels in the culture supernatant of KLE cells (Figure 5), as measured by ELISA. These findings are especially important in the context of endometrial cell proliferation and activation, since MMP-2, MMP-9, and VEGF are key regulators of inflammatory and fibrotic processes in the endometrium.

## 3. Discussion

The present study shows for the first time that Miodesin^TM^ presents anti-inflammatory effects, inhibiting hyperactivation of endometrial epithelial cell KLE and vaginal epithelial cell VK2 E6/E7, which are cells presenting a key role in the pathophysiology of endometriosis and leiomyoma [2,3]. Such effects of Miodesin^TM^ were evidenced by inhibition in the cytokines (IL-6, IL-8, IL-1β, and TNF-α) and chemokine release (CCL2/MCP-1; CCL3/MIP-1α), beyond the inhibition of other inflammatory (COX-1, COX-2, PLA2) and pro-fibrotic (MMP-2, MMP-9, and VEGF) mediators. In addition, Miodesin^TM^ efficiently inhibited the transcription factor NF-kβ.

Endometriosis is still a disease with uncertain pathophysiology. However, the inflammatory character associated with the role of cytokines, hormones, and soluble factors seems to play an important role in its pathophysiology [28]. Indeed, the inflammatory character in endometriosis has been studied in the peritoneal fluid and serum, in addition to the eutopic and ectopic endometrium [29,30,31,32]. In this way, increased levels of TNF-α, IL-6, IL-8, IL-10, VEGF, and MCP-1/CCL2 [29,32,33], as well as different metalloproteinases (MMPs), are associated with the establishment and progression of endometriosis [34]. In fact, the present study revealed that vaginal mucosa and endometriotic cells responded to increased levels of IL-6, IL-8, IL-1β, and TNF-α when challenged by *Candida albicans* or by LPS, and these cells present a pivotal role in the inflammatory process in endometriosis and leiomyoma.

On the other hand, in 2018 and 2019, Maia et al. published the first manuscripts showing the role of Miodesin^TM^ in reducing pelvic pain in patients with endometriosis and leiomyoma, supporting the role of naturally occurring anti-inflammatory medications in the treatment of this disease [8,26,27]. However, no analysis concerning the anti-inflammatory effects of Miodesin^TM^ was addressed in such studies. So, the present study shows for the first time that Miodesin^TM^ (10 µg/mL and 200 µg/mL) inhibited the hyperactivation of vaginal mucosa and endometriotic cells driving an inflammatory response, as demonstrated by reduced release of cytokines (IL-1β, IL-6, IL-8 and TNF-α; *p* < 0.01) and chemokines (CCL2, CCL3 and CCL5; *p* < 0.01)—in addition to the reduction on the expression at mRNA levels of transcription factor (NF-kβ; *p* < 0.01), inflammatory enzymes (COX-1, COX-2, PLA2, iNOS; *p* < 0.01), and chemokines (CCL2, CCL3 and CCL5; *p* < 0.01). Of note, the cytokines inhibited by Miodesin^TM^, such as IL-8 [32,35], IL-6 [36,37], IL-1β [38,39], and TNF-α [40,41] are thought to have a key role in the inflammatory process in endometriosis, becoming an interesting tool as an adjuvant for therapeutic schemes in the treatment of endometriosis. Thus, the inhibitory effects of Miodesin^TM^ on epithelial cells in the human lower female genital tract clearly showed its anti-inflammatory potential, ensuring further randomized clinical trials.

Although inflammation plays a pivotal role in endometriosis, which is regulated by pro-inflammatory cytokines [42,43], it is important to note the modulatory capacity of Miodesin^TM^ reducing the release of IL-8 by vaginal mucosa and endometriotic cells. IL-8 is also considered a chemokine, which is involved in the pathogenesis of endometriosis [44]. Chemokines are proteins with the ability to control mainly immune cell chemoattraction, but also centrally involved in cell proliferation, migration, and apoptosis [45]. In the present study, stimulation with *C. albicans* induced a significant increase in the secretion of chemokines CCL2, CCL3, and CCL5 by vaginal mucosa cells. Again, treatment with Miodesin^TM^ resulted in significant reduction in CCL2, CCL3, and CCL5 expression at mRNA level and only for CCL2 and CCL3 at protein levels. In addition, chemokines form a complex network and stimulate other cells, such as macrophages, stromal cells, epidermal cells, and smooth muscle cells [46]. Recent studies have shown several types of chemokines produced by the endometrial implant foci, thus affecting the progression of endometriosis [46]. In this sense, both IL-8 and CCL2 are elevated in endometriotic tissues and in peritoneal implants, reinforcing the role of chemokines in the progression of ovarian and peritoneal endometriosis [47]. CCL5 is another example of a chemokine found high in the peritoneal fluid of women with endometriosis and is proportional to the stage of the disease that seems to mediate the chemotactic activity of monocytes in the peritoneal cavity, suggesting that it may contribute to the progression of this disease [48]. Thus, in this study we also evaluated the behavior in the secretion of chemokines CCL2, CCL3, and CCL5 in the KLE endometrial cell line. The results showed, once again, the ability of Miodesin^TM^ to reverse the increase in chemokine secretion, when endometrial cells were stimulated by LPS, reinforcing the therapeutic potential of Miodesin^TM^ in therapeutic endometriosis treatment schemes.

Furthermore, in endometrial cells, we found that the secretion and expression of the mRNA levels of the metalloprotease enzymes MMP-2 and MMP-9 and of growth factor VEGF were reduced. The results showed that treatment with Miodesin^TM^ led to a significant reduction in the secretion of MMP-2, MMP-9, and VEGF by KLE cells, measured at protein levels. Similarly, such inhibitory effects of Miodesin^TM^ were also seen at mRNA levels. Studies suggest that MMPs are associated with the establishment and progression of endometriosis and their levels appear to be increased in ectopic endometriotic tissues [5]. MMP-2 and MMP-9 have been extensively studied in the scope of endometriosis, mainly because they are present at high levels in the peritoneal fluid of patients with endometriosis [49]. In addition, MMP-2 and MMP-9 are found to have high levels and activity in human endometriotic tissues [50]. Downregulation of MMP-2 and MMP-9 by Miodesin^TM^ may involve the inhibition of migration and invasion in human endometriotic cells, considering that MMPs have been implicated in this phenomenon. VEGF is one of the main growth factors in angiogenesis and since endometriosis is characterized by marked vascularization in and around ectopic tissue, VEGF-induced angiogenesis may be a critical aspect of the pathophysiology of endometriosis [46].

Finally, in addition to VEGF, chemokines and cytokines can play important roles in angiogenesis associated with endometriosis. IL-8, for example, a chemoattractant for neutrophils, is an angiogenic agent, inducing proliferation of endothelial cells [51]. TNF-α, which induces the growth of new blood vessels and secreted by macrophages, also stimulates the proliferation of endometriotic stromal cells [52].

## 4. Materials and Methods

### 4.1. Reagents

Dulbecco’s modified Eagle’s medium (DMEM), keratinocyte serum-free medium, fetal bovine serum (FBS), penicillin, streptomycin, and phosphate-buffered saline (PBS) were obtained from Gibco BRL (Grand Island, CA, USA). 3-(4,5-Dimethylthiazol-2-yl)-2,5-diphenyltetrazolium bromide (MTT) was purchased from Sigma-Aldrich (St. Louis, MO, USA). The Miodesin™ was supplied by Fagron™ (São Paulo, Brazil). The purity and quality of the raw materials used, as well as the formulation of Miodesin™ (*Uncaria tomentosa*, *Endopleura uchi* and Astaxanthin) were monitored by the Fagron™ Brazil quality control department.

### 4.2. Culture and Cytotoxicity Evaluation of Miodesin by MTT Assay

Endometrial cell line KLE (epithelial) and vaginal cell line VK2 E6/E7 (epithelial) were cultured in Dulbecco’s modified Eagles’ medium (DMEM high glucose) supplemented with 10% *v*/*v* fetal bovine serum (FBS), 1% L-glutamine, 100 U/mL penicillin and 100 mg/mL streptomycin, and in keratinocyte serum-free medium (Gibco) supplemented with 50 µg of bovine pituitary extract, 0.1 ng of epidermal growth factor, 100 U of penicillin, and 100 µg of streptomycin, respectively and maintained at 37 °C in a humidified atmosphere of 5% CO_2_. The cells were trypsinized every 72 h using 0.01% trypsin and 1 mmol ethylenediamine tetra-acetic acid (EDTA). For all the experiments, the Miodesin™ was dissolved in the culture medium in appropriate concentrations. The cell viability of control and Miodesin^TM^ (1–1.000 μg/mL)-treated cells were measured using a standard MTT assay. Briefly, 5 × 10^4^ viable cells were seeded into clear 96-well flat-bottom plates (Corning) in RPMI medium supplemented with 10% fetal bovine serum and incubated with different concentrations of the extract for 24 h. Then, 10 μL/well of MTT (5 mg/mL) was added and the cells were incubated for 4 h. Following incubation, 100 μL of 10% sodium dodecyl sulfate solution in deionized water was added to each well and left overnight. The absorbance was measured at 595 nm in a benchtop multimode reader (Molecular Device). The established cell lines KLE and VK2 E6/E7 were kindly provided by Dr. Andre Luis Lacerda Bachi, from University Santo Amaro, São Paulo, SP, Brazil.

### 4.3. Candida Albicans (Stimulator Cells)

The isolate of *Candida albicans* was grown on Sabouraud dextrose agar (Becton Dickinson, Cockeysville, MD, USA) at 30 °C, and one colony was used to inoculate 10 mL of Phytone-peptone broth (Becton Dickinson) supplemented with 0.1% glucose. Broth cultures were grown to stationary phase for 18 h at 25 °C in a shaking water bath. The blastoconidia were collected, washed with phosphate-buffered saline, and enumerated on a hemacytometer by using trypan blue dye exclusion.

### 4.4. Coculture Supernatant Collection for Examination of Cytokines and Chemokines

For examination of cytokines and chemokines, epithelial cells (1 × 10^5^ to 5 × 10^5^ cells/mL) were cocultured with *Candida albicans* (1 × 10^7^ to 5 × 10^7^/mL) at a ratio of 1:100 in separate wells for 0, 24, 48, and 72 h for VK2 E6/E7 cells in a total volume of 2 mL of tissue culture medium in 24-well tissue culture plates (Costar, Corning, NY, USA). Controls included VK2 E6/E7 cells (constitutive production) cultured alone in separate wells, *Candida albicans* cultured alone (negative control), and cells cultured with tumor necrosis factor alpha (TNF-alpha; 10 to 20 ng/mL [quality control]; Pharmingen, San Diego, CA, USA) independently in tissue culture medium alone over the same 96-h period. At each time point, the VK2 E7/E6 cell-Candida coculture and the respective control cultures were aspirated from a new set of individual wells and centrifuged for 5 min at 800× *g*. Thereafter, the supernatants were collected and stored at −70 °C until assayed.

### 4.5. Cytokine and Chemokine Analysis of Coculture and Endometrial Cells Supernatants

The concentrations of TNF-α, IL-6, IL-8, IL-1β, MCP-1 (CCL2), MIP-1-α (CCL3), and RANTES (CCL5), in the supernatants obtained from different groups were analyzed using enzyme-linked immunosorbent assay (ELISA) kits (R&D Systems, Minneapolis, MN, USA) following the manufacturer’s instructions. For endometrial cell line cells, they were treated with LPS (1 μg/mL) with or without Miodesin (10 µg/mL and 200 μg/mL) for 6 h. The culture supernatant (100 μL) was removed to determine the level of cytokines and chemokines, according to the manufacturer’s instructions.

### 4.6. Reverse Transcription-Quantitative PCR (RT-qPCR)

Total RNA extracted from cells samples was converted to cDNA using a SuperScript^®^ III RT kit (Invitrogen, Carlsbad, CA, USA), according to the manufacturer’s protocol. The concentration of RNA was detected using a NanoDrop 2000 (Thermo Fisher Scientific, Inc., Waltham, MA, USA). GAPDH and 18 S rRNA were used as the internal control. The thermocycling conditions were as follows: 95 °C for 10 min followed by 35 cycles of 95 °C for 15 s and 55 °C for 40 s. The 2^−ΔΔCq^ method was used to quantify the relative gene expression levels of the target genes. Relative standard curves were generated by serial dilutions and all samples were run in triplicates. The table below indicates the sequences of primers used in qRT-PCR analysis.
**Gene****Primer sequences**NF-κβForward 5′-ATGGCTTCTATGAGGCTGAG- 3′Reverse 5′-GTTGTTGTTGGTCTGGATGC- 3′COX-1Forward 5′-AGGAGATGGCTGCTGAGTTGG-3′Reverse 5′-AATCTGACTTTCTGAGTTGCC-3′COX-2Forward 5′-ACACCTTCAACATTGAAGACC-3′ Reverse 5′-ATCCCTTCACTAAATGCCCTC-3′PLA2Forward 5′-AAAGAACACTATAGGGAGAG-3′Reverse 5´-AAAGAGGTAAAGGGCATTGT-3′ CCL2Forward 5′-GATCCCAATGAGTAGGCTGG-3′Reverse 5′-CGGGTCAACTTCACATTCAAAG-3′CCL3Forward 5′-ACACCAGAAGGATACAAGCAG-3′Reverse 5′-CGATGAATTGGCGTGGAATC-3′CCL5Forward 5′-CCCACGTCAAGGAGTATTTCTAC-3′ Reverse 5′-CTAGGACTAGAGCAAGCGATG-3′MMP-2Forward 5′-ACCGCGACAAGAAGTATGGC-3′Reverse 5′-CCACTTGCGGTCA TCATCGT-3´MMP-9Forward 5′-CGATGACGAGTTGTGGTCCC-3′Reverse 5′-TCGTAGTTG GCCGTGGTACT-3′VEGFForward 5′-TGCAGATTATGCGGATCAAACC-3′Reverse 5′-TGCATTCACATTTGTTGTGCTGTAG-3′GAPDHForward 5′-CGGTGTGAACGGATTTGGC-3′Reverse 5′-GTGAGTGGAGTCATACTGGAAC-3′

### 4.7. Enzyme-Linked Immunosorbent Assay for the Determination of MMP2 and MMP9

Cell supernatant was centrifuged at 12,000× *g* for 15 min at 4 °C. MMP2 ELISA kits (RAB0365; Sigma, San Louis, MO, USA) and MMP9 ELISA kits (RAB0372; Sigma, Nanjing, China) were used to measure the levels of protein secretion of MMP2 and MMP9 by glioma cells, respectively, according to the manufacturer’s instructions. To detect VEGF concentration in supernatants, ELISA was performed with a commercially available ELISA kit (Abcam, Cambridge, UK). The VEGF concentrations were detected according to the manufacturer’s instructions.

### 4.8. Statistical Analysis

The unpaired Student’s *t* test was used to analyze constitutive and Candida-specific cytokine production between primary oral and vaginal epithelial cells. Significant differences were defined as having a *p* value ≤ 0.05. One-way analysis of variance (ANOVA) was used for multiple comparisons. The obtained results were expressed as the mean ± standard error of mean from at least three independent experiments, unless stated otherwise.

## 5. Conclusions

Miodesin^TM^ exerts anti-inflammatory effects against *C. albicans* and LPS stimulation, inhibiting vaginal mucosa and endometriotic cells hyperactivation. The results show that Miodesin^TM^ is a promising phytotherapeutic candidate for treatment of endometriosis and leiomyoma.

## Figures and Tables

**Table 1:** Effects of Miodesin^TM^ on the cytokine secretion by vaginal cell line (VK2 E6/E7). Cells were stimulated with *Candida albicans* in the presence of Miodesin^TM^ (10 µg/mL and 200 µg/mL) and cultured for 24 h. Cytokines were determined in the culture supernatants by ELISA. Representative data obtained in one of three different experimental series are shown. Mean values ± SEM (of triplicate cultures) are given. (^#^) *p* < 0.05. (*Candida albicans* vs. medium). (*) *p* < 0.05. (*Candida albicans*-stimulated cells + Miodesin^TM^ vs. *Candida albicans*-stimulated cells).

Cytokine	Stimulus	Cytokine Concentration (Mean pg/mL ± SEM)		
		24 h	48 h	72 h
IL-6	Medium	4.9 ± 0.8	7.8 ± 2.4	8.2 ± 2.6
	*C. albicans*	17.1 ± 1.0 ^#^	31.0 ± 2.9 ^#^	64.3 ± 3.7 ^#^
	*C. albicans* + Miodesin^TM^ (10 µg/mL)	6.2 ± 2.3 *	12.9 ± 3.8 *	28.2 ± 3.9 *
	Medium	3.4 ± 0.5	12.6 ± 2.8	26.0 ± 3.7
	*C. albicans*	19.0 ± 0.9 ^#^	39.0 ± 3.7 ^#^	68.0± 4.5 ^#^
	*C. albicans* + Miodesin^TM^ (200 µg/mL)	11.0 ± 2.9 *	15.0 ± 3.5 *	29.0 ± 3.2 *
IL-8	Medium	31.4 ± 2.4	35.2 ± 2.9	32.1 ± 3.2
	*C. albicans*	63.0 ± 4.0 ^#^	76.6 ± 3.0 ^#^	102.4 ± 3.7 ^#^
	*C. albicans* +Miodesin^TM^ (10 µg/mL)	33.9 ± 2.7 *	26.8 ± 1.9 *	29.7 ± 3.1 *
	Medium	30.0 ± 3.7	31.3 ± 1.6	29.6 ± 3.8
	*C. albicans*	69.0 ± 5.1 ^#^	87 ± 2.5 ^#^	107.6 ± 3.4 ^#^
	*C. albicans* + Miodesin^TM^ (200 µg/mL)	36.0 ± 3.5 *	21 ± 2.9 *	20.3 ± 1.7 *
IL-1β	Medium	4.7 ± 2.6	5.8 ± 2.1	5.7 ± 1.4
	*C. albicans*	15.3 ± 2.3 ^#^	23.2 ± 2.8 ^#^	31.5 ± 3.3 ^#^
	*C. albicans* + Miodesin^TM^ (10 µg/mL)	9.0 ± 1.6 *	11.5 ± 2.5 *	17.3 ± 1.1 *
	Medium	3.3 ± 2.1	3.9 ± 1.8	6.0 ± 2.3
	*C. albicans*	18.0 ± 1.9 ^#^	22.0 ± 3.9 ^#^	32.0 ± 3.1 ^#^
	*C. albicans* + Miodesin^TM^ (200 µg/mL)	12.0 ± 1.1 *	17.0 ± 3.5 *	18.0 ± 2.1 *
TNF-α	Medium	7.1 + 0.9	9.2 + 3.2	8.6 + 1.7
	*C. albicans*	25.2 + 2.1 ^#^	29.9 + 4.1 ^#^	37.0 + 3.2 ^#^
	*C. albicans* + MiodesinTM (10 µg/mL)	11.1 + 1.9 *	13.2 + 2.1 *	14.9 + 4,2 *
	Medium	5.7 ± 0.2	12.6 ± 2.8	18.6 ± 3.7
	*C. albicans*	21.5 ± 0.5 ^#^	31.9 ± 3.7 ^#^	67.2 ± 2.5 ^#^
	*C. albicans* + Miodesin^TM^ (200 µg/mL)	11.0 ± 2.7 *	16.0 ± 3.5 ^*^	31.0 ± 3.2 *

**Table 2:** Effects of Miodesin^TM^ on the chemokine secretion by vaginal cell line (VK2 E6/E7). Cells were stimulated with *C. albicans* in the presence of Miodesin^TM^ (10 µg/mL and 200 µg/mL) and cultured for 24 h. Chemokines were determined in the culture supernatants by ELISA. Representative data obtained in one of three different experimental sets are shown. Mean values ± SEM (of triplicate cultures) are given. (^#^) *p* < 0.05. (*Candida albicans* vs. medium). (*) *p* < 0.05. (*Candida albicans*-stimulated cells + Miodesin^TM^ vs. *Candida albicans*-stimulated cells).

Cytokine	Stimulus	Cytokine Concentration (Mean pg/mL ± SEM)		
		24 h	48 h	72 h
CCL2 (MCP-1)	Medium	3.3 ± 2.9	4.7 ± 1.2	6.5 ± 2.8
	*C. albicans*	10.1 ± 2.2 ^#^	14.2 ± 2.9 ^#^	21.5 ± 1.5 ^#^
	*C. albicans* + Miodesin^TM^ (10 µg/mL)	6.7 ± 3.2 *	8.9 ± 3.5 *	10.1 ± 1.1 *
	Medium	2.1 ± 3.2	4.9 ± 2.2	8.10 ± 1.7
	*C. albicans*	9.8 ± 1.2 ^#^	16.0 ± 3.2 ^#^	31.0 ± 2.5 ^#^
	*C. albicans* + Miodesin^TM^ (200 µg/mL)	5.2 ± 1.1 *	12.6 ± 1.5 *	19.8 ± 1.7 *
CCL3 (MIP-1α)	Medium	33.0 ± 2.8	35.2 ± 3.9	31.4 ± 2.3
	*C. albicans*	58.6 ± 3.3 ^#^	69.8 ± 4.1 ^#^	75.2 ± 2.8 ^#^
	*C. albicans* + Miodesin^TM^ (10 µg/mL)	27.1 ± 2.5 *	31.6 ± 1.9 *	25.2 ± 3.2 *
	Medium	30.0 ± 3.7	32.3 ± 1.6	29.6 ± 3.8
	*C. albicans*	59.0 ± 3.1 ^#^	67 ± 2.5 ^#^	81.7 ± 3.4 ^#^
	*C. albicans* + Miodesin^TM^ (200 µg/mL)	26.0 ± 3.5 ^*^	29 ± 2.9 *	24.0 ± 1.7 *
CCL5 (RANTES)	Medium	3.7 ± 1.9	3.1 ± 2.8	3.6 ± 1.7
	*C. albicans*	19.1 ± 3.7 ^#^	24.2 ± 2.7 ^#^	29.9 ± 2.9 ^#^
	*C. albicans* + Miodesin^TM^ (10 µg/mL)	9.2 ± 4.5	11.9 ± 2.5	12.8 ± 5.8
	Medium	3.3 ± 2.1	3.9 ± 1.8	4.0 ± 2.3
	*C. albicans*	16.0 ± 2.9 ^#^	22.0 ± 3.9 ^#^	32.0 ± 3.1 ^#^
	*C. albicans* + Miodesin^TM^ (200 µg/mL)	9.3 ± 4.1	19.9 ± 3.9	28.0 ± 5.5

**Table 3:** Effects of Miodesin^TM^ on the chemokine secretion by endometrial cell line (KLE). Cells were stimulated with LPS in the presence of Miodesin^TM^ (10 µg/mL and 200 µg/mL) and cultured for 24 h. Chemokines were determined in the culture supernatants by ELISA. Mean values ± SEM (of triplicate cultures) are given. (^#^) *p* < 0.05. (LPS-stimulated cells vs. medium). (*) *p* < 0.05. (Miodesin^TM^ vs. LPS-stimulated cells).

Cytokine	Stimulus	Cytokine Concentration (Mean pg/mL ± SEM)		
		24 h	48 h	72 h
CCL2 (MCP-1)	Medium	7.1 ± 2.1	6.2 ± 2.2	7.9 ± 4.1
	LPS	15.2 ± 3.1 ^#^	17.2 ± 3.6 ^#^	21.3 ± 3.7 ^#^
	LPS + Miodesin^TM^ (10 µg/mL)	6.1 ± 1.4 *	8.9 ± 3.3 *	9.2 ± 1.1 *
	Medium	4.2 ± 2.9	6.4 ± 3.9	11.3 ± 3.2
	LPS	13.3 ± 3.9 ^#^	21.4 ± 5.1 ^#^	42.2 ± 3.5 ^#^
	LPS + Miodesin^TM^ (200 µg/mL)	6.9 ± 2.4 *	12.3 ± 2.3 *	19.1 ± 2.1 *
CCL3 (MIP-1α)	Medium	33.2 ± 1.7	39.4 ± 2.2	36.3 ± 3.7
	LPS	67.2 ± 3.8 ^#^	75.2 ± 2.9 ^#^	81.1 ± 6.2 ^#^
	LPS + Miodesin^TM^ (10 µg/mL)	31.2 ± 4.2 *	33.8 ± 5.2 *	29.8 ± 11.1 *
	Medium	31.1 ± 2.2	42.1 ± 3.7	31.2 ± 5.8
	LPS	69.1 ± 2.9 ^#^	77.8 ± 3.8 ^#^	93.2 ± 8.9 ^#^
	LPS + Miodesin^TM^ (200 µg/mL)	21.1 ± 4.5 *	31.2 ± 6.9 *	20.4 ± 10.7 *
CCL5 (RANTES)	Medium	5.2 ± 4.1	8.4 ± 2.5	7.4 ± 2.8
	LPS	30.3 ± 3.1 ^#^	36.2 ± 4.1 ^#^	39.6 ± 3.7 ^#^
	LPS + Miodesin^TM^ (10 µg/mL)	9.4 ± 6.2 *	11.3 ± 4.1 *	12.9 ± 4.7 *
	Medium	4.4 ± 7.9	3.9 ± 1.8	6.7 ± 2.3
	LPS	29.9 ± 5.1 ^#^	32.0 ± 3.9 ^#^	41.0 ± 3.1 ^#^
	LPS + Miodesin^TM^ (200 µg/mL)	13.5 ± 8.1 *	18.1 ± 5.2 *	21.8 ± 4.9 *

## Data Availability

All raw data will be available upon a reasonable justification request.

## Sample Availability

Samples of the compounds will be available upon a reasonable justification request.
